# Polymorphic Transformations of Pharmaceutical Materials Induced by Mechanical Milling: A Review

**DOI:** 10.3390/pharmaceutics17070946

**Published:** 2025-07-21

**Authors:** Mathieu Guerain, Jean-François Willart

**Affiliations:** Université de Lille, CNRS, INRA, ENSCL, UMR8207, UMET, Unité Matériaux et Transformations, F-59650 Villeneuve d’Ascq, France; mathieu.guerain@univ-lille.fr

**Keywords:** pharmaceuticals, milling, polymorphic transformations, amorphization, molecular alloys, glass transition

## Abstract

A review of the literature on polymorphic transformations by milling on pharmaceutical materials was carried out. The available information on 18 pharmaceutical materials was compiled. In particular, when data are available, the starting and final crystalline forms, their enantiotropic or monotropic relationship, the glass transition temperature of the compound and its melting temperature, the experimental observation of a transient or partial amorphization of compounds, and the transformation kinetics make it possible to suggest a two-step transformation mechanism. First, an amorphization occurs under milling of the starting polymorphic form. Secondly, a recrystallization of the amorphous form occurs towards the final form. The observed transformation kinetics are due to the fact that the recrystallization of the amorphous material towards the final form depends on the accidental formation of a cluster of this form during milling. Moreover, the observation of the transient amorphous form depends on the relative position of the glass transition temperature of the material with respect to the milling temperature. This mechanism seems to be independent of the enantiotropic or monotropic character of the polymorphic forms involved in the transformation.

## 1. Introduction

The physical and pharmaceutical properties (physical and chemical stability, solubility, tabletability, and bioavailability, for example) of pharmaceutical molecular materials are directly influenced by their structural state (amorphous, crystalline) and, in particular, by their polymorphic form in the crystalline state [1]. It is therefore crucial to identify and to use the most efficient polymorphic form of a given active principle ingredient (API) and to monitor any potential transformations that may occur during the different stages of the drug manufacturing process.

Milling is a process frequently used in the pharmaceutical industry to reduce the particle size of powders which generally improve dissolution kinetics, flowability, compaction, and the accuracy of dosages. However, long-term and high-energy millings can also induce various structural transformations, such as amorphization [2,3,4,5,6,7,8] (e.g., trehalose [8], lactose [2], dexamethazone [6], linaprazan [4], and griseofulvine [7]) or polymorphic transformations [9,10] (e.g., glycine [11], sorbitol [12,13], mannitol [14]). These transformations must be carefully controlled as they can modify the therapeutic efficiency of the drug as well as its physical and chemical stability.

The influence of the glass transition temperature (T_g_) on the structural state of the compound obtained after milling is well known. It is well established that when the milling temperature (T_mill_) is lower than the glass transition temperature of the compound, an amorphization is generally observed [15]. On the other hand, when the T_mill_ is higher than the T_g_ of the material, we can observe either an absence of physical transformation (except a reduction in the size of the crystallites), or a polymorphic transformation of the compound [15]. While several examples of polymorphic transformations of various pharmaceutical compounds are reported in the literature, the mechanisms which drive these polymorphic transformations remain poorly understood. In particular, it is not clear if these polymorphic transformations induced by milling are achieved by direct displacements of molecules in the crystallographic lattice or if they involve, on the contrary, a transient amorphization of the initial crystalline form followed by an extremely rapid recrystallization to a new final form [16]. It is also possible that other parameters that have been little studied could have an influence on the underlying mechanisms. The transformation mechanism could, for example, depend on the monotropic or enantiotropic relationship between the two polymorphs as it determines the existence of an equilibrium phase transition between the two forms. However, the transformation mechanism could also depend on the difference between the T_mill_ and T_g_, the relative stability of the final and starting forms, or the evolution of crystallite sizes during milling.

The aim is here to enlighten the mechanisms of polymorphic transformations induced by milling through a careful analysis of the literature and targeted experiments on a number of compounds that exhibit polymorphic transformations under milling. This work will be divided in two papers. In the first one, we will review the literature which report pharmaceutical materials presenting such transformations. The observations and conclusions of the different authors on this subject will be discussed to take stock of the understanding of these polymorphic transformation mechanisms. On the other hand, in a second paper, we will carry out different milling experiments on several compounds chosen to complete and give relevant information to the conclusions drawn in the first paper. Both papers will lead to propose a general mechanism of polymorphic transformation under milling.

In this first paper, we have searched for and analysed all the polymorphic transformations of pharmaceutical compounds that we were able to find in the literature. In order to better understand and rationalize these transformations, when the information was available, we identified the T_g_ of the material, the stable or metastable nature of the starting and final forms, the relationships (enantiotropic or monotropic) between the different polymorphs, and the number of polymorphs known in the literature; the changes in crystallite sizes during milling is also discussed. It must also be noted that the kinetics of polymorphic transformations upon milling are the direct reflection of the microscopic mechanisms which govern these transformations. As a result, a detailed and careful investigation of these kinetics is thus expected to help the understanding of these solid-state transformations. So far, these transformations kinetics have been very little studied, and only a few examples have been reported in the literature. Special attention is thus paid to materials whose polymorphic transformation upon milling has been studied in the literature. Great importance is also given to the detection or not of an amorphous form of the material during the polymorphic transformation. Indeed, in the mechanisms currently proposed, it is considered that either the impacted crystal undergoes a transformation towards another crystalline form directly upon impact, or the impact causes a local amorphization followed by a recrystallization towards another polymorphic form [16]. The detection of an amorphous form will drive the observed transformations towards the second hypothesis.

## 2. Compounds with Polymorphic Transformations Reported in Literature

The list of pharmaceutical materials presented in this section provides a state of the art of polymorphic transformations under milling to lead to an explanation of the mechanisms underlying this phenomenon. The study of all these different compounds and the information obtained should make it possible to describe, as best as possible, the phenomena involved during polymorphic transformations.

### 2.1. Sorbitol

Sorbitol is a naturally occurring polyol that has a stable γ (orthorhombic) form and five polymorphic forms identified in the literature [17]. The form γ of sorbitol transforms into the metastable α (orthorhombic) form after 3 h of milling at room temperature. This transformation occurs, as expected, above its glass transition temperature (T_g_ = −3 °C) [12,18]. The experiments carried out by Dupont et al. [12,13] suggest a transformation mechanism involving the passage through an intermediate amorphous compound between the γ and α phases. Amorphization of sorbitol at room temperature was also observed: when the γ form of sorbitol was mixed with an amorphous compound (hydrochlorothiazide, previously amorphized), the γ form of sorbitol amorphized largely before transforming into the α form.

### 2.2. Bezafibrate

Bezafibrate is used in the treatment of hypercholesterolemia and hypertriglyceridemia. It is a poorly soluble active ingredient (0.016 ± 0.003 mg.mL^−1^) with two crystalline forms: α and β [19]. These two forms have an enantiotropic relationship. The β form is the stable form at room temperature, and a solid–solid transition to the α form takes place around 160 °C. This α form then melts at 185 °C. The glass transition temperature of Bezafibrate is 37 °C. At room temperature, milling of the α form leads to the β form after less than 3 h [20]. Some amorphous (~10%) form is observed during this transformation. On the other hand, at −10 °C, milling of Bezafibrate α leads to an amorphous compound [20].

### 2.3. Sulfamerazine

Sulfamerazine is an antibacterial agent with three polymorphic forms, noted as form I [21], form II [22], and form III [23]. Forms I and II have an enantiotropic relationship. form II is stable at room temperature, and form I is stable at high temperatures [24]. Cryomilling of forms I and II systematically leads to amorphization of the compound. On the other hand, at room temperature, form I is transformed into a mixture of form II and amorphous in less than an hour of milling. Form II, on the other hand, is not transformed, and only a reduction in the size of the crystallites is observed without quantification [25]. The glass transition temperature of Sulfamerazine is 62 °C [25], which is only slightly higher than room temperature. For this reason, the heating produced in the jar by the impact of the balls can lead to temperatures more or less close to this glass transition temperature (depending on the milling conditions), which can change the nature of the transformation.

### 2.4. Mannitol

Mannitol, is an acyclic sugar alcohol produced by various plants, algae, and fungi and is mainly used in the pharmaceutical industry as an excipient in the formulation of tablets and granulated powders. Mannitol has three polymorphic forms δ, α and β [26,27]. Milling the β form of mannitol (the most stable) leads to its transformation to the α form in several hours according to the works of Martinetto et al. [14]. In this study, the evolution of the crystallite sizes during the transformation was monitored. Calculations showed a sudden decrease in size during the first 5 min of milling and then a steady state, while the transformation really began after 90 min of milling. The glass transition temperature of mannitol is 13 °C [28], which is slightly lower than room temperature.

### 2.5. Glycine

Glycine is the smallest of the amino acids and is a precursor of many proteins such as creatine, uric acid, and acetylcholine. Glycine is used in the food industry as a sweetener (E640) or flavor enhancer. It has three known polymorphic forms: α, β, and γ [29]. The γ form (trigonal) transforms into the α form (monoclinic) during milling at room temperature [11]. The melting temperature of glycine is still very poorly defined because this compound degrades upon heating well before melting. It is evaluated by theoretical or indirect approaches between 160 and 423° [30,31,32,33,34,35,36]; also, the glass transition temperature has never been able to be obtained or evaluated precisely. The amorphization of glycine has, so far, never been observed in the literature.

### 2.6. Sulfathiazole

Sulfathiazole is an antimicrobial that has five known polymorphic forms denoted from I to V [37,38]. According to the work of Shakhtshneider and Boldyrev [39], it is possible to transform form III into form I after 90 min of milling, but it is also possible to transform form I into form III after an equivalent milling time. It is noted that the formation of an amorphous phase during the first moments of milling plays an important role during the transformation. The work of Hu et al. [40] demonstrated that starting from any of the five forms, milling takes two hours to a mixture of form I and amorphous form in the absence of solvents. The glass transition temperature is 67 °C [41].

### 2.7. Ranitidine Hydrochloride

Ranitidine hydrochloride is an antihistamine used in the treatment of stomach ulcers. This compound has two known polymorphic forms, forms I [42] and II [43], both monoclinic with monotropic relationship. Form II is considered the more stable form because it has a higher melting temperature [44]. Forms I and II were milled by Chieng et al. [45] for approximately 4 h at 4, 12, and 35 °C. They measured that these temperatures corresponded to temperatures in the jar of 36, 45, and 62 °C, respectively. In the first case (ambient temperature of 4 °C; therefore, 36 °C in the jar), it was observed that the milling of form I produced only an amorphous form. In the other two cases, a transformation from form I to form II via an amorphous form was observed. Form II was stable under milling. The glass transition temperature of the compound is between 15 and 23 °C when milling is performed at room temperature [45] and at 35 °C, and it is between 13 and 30 °C when milling is performed at 4 °C.

### 2.8. Rivastigmine Hydrogen Tartrate

Rivastigmine hydrogen tartrate (RHT) is used in the treatment of dementia due to Alzheimer’s disease. It has two polymorphic forms [46] noted: form I [47] (monoclinic) and form II, whose structure is not known. The two forms have a monotropic relationship, with form I being the stable form [46]. Milling of the commercial form (form II) leads to a transformation into form I after several hours according to Amaro et al. [46]. It is also clearly shown that the amorphous form is produced by milling form II, but due to the low glass transition temperature of the compound, it quickly recrystallizes to form I [46]. The glass transition temperature of rivastigmine hydrogen tartrate is 38.2 °C [46] when obtained by melt quenching.

### 2.9. Famotidine

Famotidine is an antihistamine used in the treatment of stomach ulcers. It has two forms that have a monotropic relationship, Forms A [48] and B [49], both monoclinic. Form B transforms into form A (the most stable form) in less than an hour of milling, according to the work of Lin et al. [50]. The glass transition temperature of famotidine is 50 °C according to Mahlin and Bergström [51].

### 2.10. Gabapentin

Gabapentin is an anticonvulsant used in the treatment of epilepsy. Gabapentin has one pseudo polymorph and three polymorphic forms. These forms are noted from I to IV. The first form is a monohydrate while the other forms—II, III, and IV—are anhydrous [52]. All forms are monoclinic [53,54]. The work of Lin et al. [52] indicated several polymorphic transformations under milling. The milling of Form I led, in about two hours, to Form II. The milling of form II led to a mixture of forms III and IV. The milling of form III led to form IV through a mixture between forms II and III. Finally, the milling of form IV led very quickly to form II (a few minutes), but this form was then converted to form III and then to IV in a few tens of minutes. The glass transition temperature of this compound is unknown.

### 2.11. Indomethacin

Indomethacin is a nonsteroidal anti-inflammatory drug used to reduce fever, pain, and inflammation. It has two polymorphic forms, the γ form (triclinic) [55], which is the most stable form, and a metastable form α (monoclinic) [56]. The work of Otsuka et al. [57,58,59] and the work of Desprez [60,61] have indicated that milling indomethacin at 4 °C leads to an amorphous form regardless of the starting form. In contrast, at 30 °C, milling the γ form leads to a transformation to the α form in about 10 h [57,58,59,60]. Milling the α form at 30 °C only leads to a reduction in the crystallite sizes of the material. The glass transition temperature of indomethacin is at 47 °C [62].

### 2.12. Modafinil

Modafinil is a psychostimulant used in the treatment of narcolepsy and hypersomnia. It has seven polymorphic forms noted I to VII [9]. Form I is the most stable form. The structures of Forms I [63] (monoclinic), III [64] (orthorhombic), and IV (orthorhombic) [65] only are known in the literature. The work of Linol et al. [9] showed that regardless of the starting form that was milled (I, III, IV, V, and VI having been tested), after about 10 h, the final form was systematically form III, when the milling was carried out without the addition of solvent. On the other hand, when a drop of water was added to carry out “wet- milling”, then form I was systematically observed. The glass transition temperature of this compound is about 43 °C (private communication).

### 2.13. Fananserin

Fananserin is a sedative and an antipsychotic that has four polymorphic forms known in the literature, denoted from I to IV [66]. Form I is triclinic, while the other are three monoclinic [66]. Form I is a metastable form, while the other three phases each have a stability domain dependent on the temperature and pressure considered. At room temperature and atmospheric pressure, phase IV is the most stable. This compound also forms a glass upon melt quenching and its glass transition temperature is 19 °C [16]. For milling times of about 30 h, this compound exhibits a transformation from forms III and IV to form I when milling is carried out at room temperature. In both cases, the authors noted the presence of a diffusion halo on the X-Ray diffractograms recorded after milling, suggesting a partial amorphization of the compound. On the other hand, when milling is carried out around 0 °C, i.e., below T_g_, the compound amorphizes [16].

### 2.14. Chloramphenicol Palmitate

Chloramphenicol palmitate is a broad-spectrum antibiotic. It has three polymorphic forms, noted A (stable, orthorhombic form), B, and C (metastable, monoclinic forms) [67,68,69]. The work of Kaneniwa and Otsuka [70] and Otsuka et al. [69] showed that milling at room temperature of the stable form A led to the amorphization of a small part of the compound. In contrast, milling of the metastable form B led to a transformation of this form into stable form A in less than 3 h. Milling of form C led to form B in less than 20 min, then this form B was transformed to 80% into form A in less than 3 h, a rate of form A which then remained stable until the end of milling (4 h). The glass transition temperature of this compound is unknown.

### 2.15. Cimetidine

Cimetidine is an antihistamine used in the treatment of ulcers and gastroesophageal reflux. It has three polymorphic forms, all monoclinic, noted A [71], B [72], and C [73] in the literature. These three forms have a monotropic relationship. The work of Bauer-Brandl [74] indicates that forms B and C (metastable) were transformed into form A (the most stable form) when they were milled. The transformation took place within 10 h in both cases. It wasalso observed that a significant but unquantified amount of amorphous material was formed. Its glass transition temperature is estimated at 43 °C [75].

### 2.16. Phenylbutazone

Phenylbutazone is a nonsteroidal anti-inflammatory drug. It has four polymorphic forms: α (monoclinic), β, δ, and ε [57,76]. The crystallographic structure of the last three forms is not known in the literature. The work of Matsumoto et al. [57] showed that the α form was transformed into the ε form after a 11 h of milling at 4 °C and into the δ form after 4 h of milling at 35 °C. The δ form was not transformed after several hours of milling at 35 °C, but after several hours of milling at 4 °C, it was transformed into the ε form. The β form was transformed into the ε form after several hours of milling at 4 °C. It was also transformed into the δ form after several hours of milling at 35 °C. The glass transition temperature of phenylbutazone is 4 °C [77].

### 2.17. Nolomirole Hydrochloride

Nolomirole hydrochloride is a molecule considered in the treatment of heart attacks. It has two polymorphic forms, α and β, whose crystallographic structures are unknown in the literature [78]. Milling for a few minutes in a mortar is enough to transform the α form into the β form. The glass transition temperature of this compound is unknown.

### 2.18. Caffeine

Caffeine acts as a psychotropic stimulant and a mild diuretic. It has two forms, form I (rhombohedral, stable at high temperatures) and form II (monoclinic, stable at room temperature), which have an enantiotropic relationship with a transition from II to I occurring at 140 °C. The work of Pirttimäki et al. [79] clearly indicates the transformation of form I to form II in less than 5 min of milling. According to the work of Mazel et al. [80], two minutes of milling allowed the transformation of form I to form II. However, the transformation was not observed immediately after milling, but 3 days later. On the other hand, when form II was milled at room temperature, it transformed into form I after less than 10 min of milling. The glass transition temperature of caffeine is estimated at −17 °C [81].

Table 1 summarizes all the information described above. In this table, the appearance of the transformation kinetics, the observation of an amorphous phase, and the relative stabilities of the phases observed before and after milling are described when this information is known. 

## 3. Analysis of the Mechanism of Polymorphic Transformation

The kinetics of polymorphic transformation under milling reflect the microscopic mechanisms involved during these transformations. The study of these kinetics is thus expected to enlighten the physical mechanisms which govern these transformations. Among the compounds listed in Table 1, a few transformation kinetics have been studied in the literature. They concern sorbitol [12,13], Bezafibrate [20], mannitol [14], glycine [11], indomethacin [85], and chloramphenicol palmitate [70]. However, the information available in the references concerning ranitidine hydrochloride [45], rivastigmine hydrogen tartrate [46], and Fananserin [16] also give an idea of the polymorphic transformation kinetics, even if the associated quantifications have not been determined.

### 3.1. Kinetics of Polymorphic Transformation Involving Two Monotropic Forms

**Figure 1:**
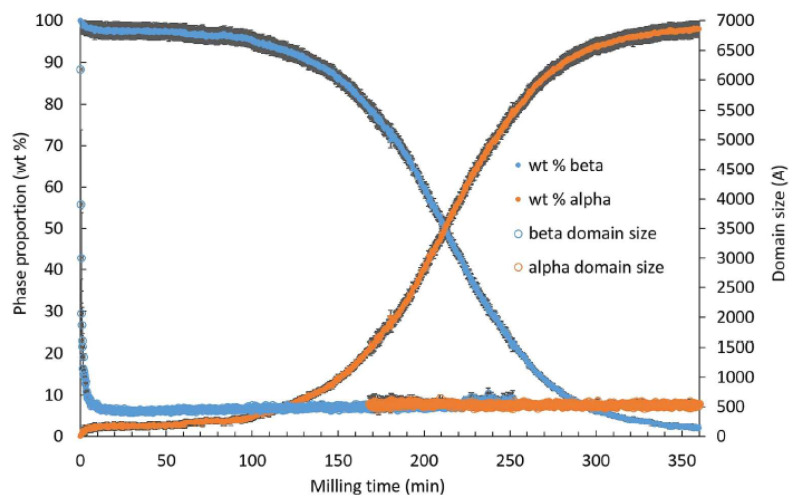
Crystalline phase proportion and coherent domain size of β-mannitol and α-mannitol. obtained by synchrotron diffraction and sequential Rietveld refinement for in situ oscillatory milling at ID15B (ESRF, Grenoble) from [14]. Reprinted with permission from [14], Copyright 2017 American Chemical Society.

Upon milling, mannitol undergoes a polymorphic transformation between the stable form β and the metastable form α, which are monotropically related. The kinetics of this transformation were studied in detail by Matinetto et al. [14]. This study took advantage of the high intensity and spatial resolution of synchrotron to follow the structural and microstructural transformations in situ, i.e., during the milling itself. The corresponding transformation kinetics is reported in Figure 1. It shows a sigmoidal shape characterized by a long incubation time of nearly two hours followed by a transformation stage of the same duration. The most striking feature of this kinetics is the rapid development of a small fraction (2.5%) of the final form α during the first two minutes of milling, which then remains constant during the whole incubation period. Similarly, a constant residual proportion (1.5%) of crystallites of the initial form β persists at the end of transformation.

These surprising kinetic characteristics could be explained within the framework of a transformation mechanism occurring in two stages: an amorphization stage caused by the impact itself, immediately followed by a recrystallization stage. The key point of the mechanism is that the nature of this recrystallization depends on the structural state of the neighboring raw crystallites. It occurs rather towards the starting form (β) if the majority of neighboring crystallites are in this form, as it is the case at the beginning of the milling process and rather towards the final form (α) otherwise. The modelling of this mechanism and its numerical simulation made it possible to reproduce the most striking characteristics of the experimental kinetics, such as the induction period and the sigmoidal shape. It also made it possible to identify and understand the four main stages of the transformation, which are:

Stage I. A relaxation towards a metastable state induced by a mechanism of creation/annihilation of α crystallites upon mechanical impacts within grains predominantly composed of γ crystallites. This stage is at the origin of the incubation period.

Stage II. A breakdown of the previous metastability caused by the accidental formation of a cluster of α crystallites. The α crystallites involved in this cluster then have several neighboring crystallites of the same nature, so that the probability that one crystallite of this cluster returns to the initial state γ after an amorphization induced by a chock becomes almost zero.

Stage III. The growth of the stable cluster formed during the previous stage due to an easier conversion to α of the impacted crystallites located in its first-neighbor layer. This ease is linked to the cluster itself, which increases the number of α-type first neighbors of the crystallites involved in this layer.

Stage IV. A relaxation towards a stationary state induced by a mechanism of creation/annihilation of a small number of β crystallites within grains mainly made of α crystallites. This situation corresponds to the exact opposite of that generated in stage I.

**Figure 2:**
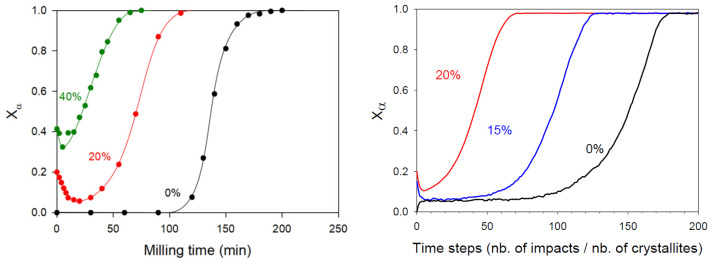
(**Left**): Experimental kinetics of γ→α transformation of sorbitol according to [12]. The left axis represents the fraction of α form. The three curves correspond to initial samples of γ-form seeded by 0, 20, and 40% of form α. The transformed fractions were derived from PXRD analysis. (**Right**): Simulated kinetics of transformation of sorbitol upon milling according to [12]. The three curves correspond to initial samples of γ-form seeded by 0, 15, and 20% of form α. A time step corresponds to 8000 impacts so that each crystallite was impacted, on average, once per unit of time. Reproduced from [12], with permission from Elsevier, 2020.

The previous mechanism of polymorphic transformation has been confirmed and strengthened by Dupont et al. [12,13], who have investigated the kinetics of the polymorphic transformation of sorbitol upon milling. This transformation drove the stable form γ towards the metastable form α, which was monotropically related to the first one. The kinetics of the transformation upon milling has been followed ex situ using conventional powder X-ray diffraction and is reported in Figure 2 (black curve). As for mannitol, the kinetics has a so-called sigmoidal shape [12,14]. It shows a long and clear induction time (about two hours), followed by a rapid transformation stage (less than an hour). Moreover, tiny traces of form α could be detected by Raman scattering during the incubation period [12]. All these kinetics features suggest that the mechanism of polymorphic transformation proposed for mannitol also applies for the polymorphic transformation of sorbitol.

The hypothesis of a metastability breaking (stage II) triggered by the accidental clustering of crystallites of the final polymorphic form was further supported by the work of Dupont et al. [12]. In their study, they monitored the kinetics of the polymorphic transformation of sorbitol γ→α after seeding the initial sample with sorbitol α. The transformation kinetics, presented in Figure 2 for the 20% and 40% seeding levels, reveal two key features that support the idea that the formation of a cluster of final α-form crystallites is necessary to initiate the transformation.

First, the incubation time associated to the transformation of the initial fraction of β crystallites decreased as the seeding fraction increased. This faster metastability breaking was attributed to the fact that the likelihood of accidentally forming a cluster of α crystallites increases with the number of pre-existing α seeds [12].

Second, the majority of the α seeds were rapidly converted to the form γ within the first few minutes of milling, indicating that isolated α crystallites—those not involved in a cluster and thus surrounded by β crystallites—were quickly transformed into the γ phase [12].

Both features thus confirm that the incubation period corresponds to the time required to form a cluster of α crystallites, which will be more stable upon milling than individual crystallites and which will facilitate the conversion of the impacted surrounding γ crystallites.

Chieng et al. evidenced a polymorphic transformation in ranitidine hydrochloride upon milling, which occurred from the metastable form I towards the stable form II [45]. While their work did not show any kinetics, strictly speaking, the diffraction patterns they observed between 0 min and 240 min of milling leads to the following conclusion: Between 0 min and 150 min of milling, no transformation from phase I to phase II was detected. After 180 min of milling, the diffraction peaks of phase I were no longer visible, while those of phase II were clearly detected. The diffraction patterns recorded at 210 min and 240 min were similar to that obtained after 180 min of milling. We therefore observed an induction time of 2 h and 30 min, followed by a rapid transformation occurring in 30 min or less (there are no intermediate points between 150 and 180 min of milling). Similar results have been obtained on rivastigmine hydrogen tartrate by Amaro et al. [46]. This material showed a transformation from the metastable form II towards the stable form I, which only started after two hours of milling and was complete after 3 h. These observations indicate that both materials were characterized by a sigmoidal kinetic of transformation.

The work of Otsuka et al. [59] on indomethacin showed that during milling at 30 °C the stable form γ was transformed into the metastable form α with an induction time of approximately 4 h, then a transformation in two hours occurred. Similar results were reported in the work of Desprez [61]. The triggering of the transformation was observed after 7 h of milling, while its completion was observed after 10 h. Here again, in both studies, the kinetics of the transformation were found to be sigmoidal.

It was also mentioned that an induction time of one hour was observed during the III→I and IV→I transformation of Fananserin in the works of De Gusseme et al. [16], although the kinetics themselves were not reported.

Moreover, in the case of monotropic materials, transitions from the stable form to the metastable form were observed during milling for sorbitol. [12,13], mannitol [14], Fananserin [16], and Modafinil [9], while the reverse was observed for ranitidine hydrochloride [45], rivastigmine hydrogen tartrate [46], famotidine [50], indomethacin [57,58,59,60], cimetidine [74], chloramphenicol palmitate [70], and phenylbutazone [57]. In both cases, a long induction time was observed. On the other hand, if the amorphous material could be observed directly for the compounds of the second group (ranitidine hydrochloride, rivastigmine hydrogen tartrate, indomethacin cimetidine), this was a priori not the case (sorbitol and mannitol) for the materials of the first group. It is therefore likely that during polymorphic transformation from the stable form to the metastable form, the latter generally forms too quickly after the transient amorphization to be detected. This can be explained by the fact that the energy difference between the amorphous phase and the stable phase is greater than between the amorphous phase and the metastable phase. In the second case, recrystallization is easier, faster, and therefore less easily detectable.

### 3.2. Kinetics of Polymorphic Transformation Involving Two Enantiotropic Forms

**Figure 3:**
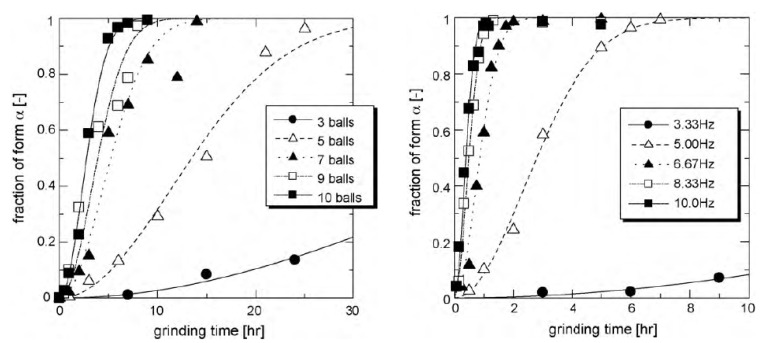
On the (**left**), time evolution of the fraction of the α form for a given milling frequency (5 Hz) but for the different numbers of balls involved. On the (**right**), time evolution of the fraction of the α form when using one milling ball but different milling frequencies (from the [11]). Reproduced from [11], with permission from Elsevier, 2010.

In the case of glycine, Matsuoka et al. [11] have shown that milling induces a polymorphic transformation from the stable form γ towards the metastable form α, these two forms being enantiotropically related. These authors have investigated the kinetics of this transformation for different milling frequencies and different numbers of milling balls, i.e., for different milling intensities. This kinetics are reported in Figure 3. As expected, the results clearly show that higher milling intensity accelerated the transformation rate. Interestingly, unlike for monotropic situations (e.g., mannitol [14] and sorbitol [12]), no distinct incubation period was observed. Instead, the transformation exhibited an acceleration stage during the first half of the kinetics, resulting in an overall S-shaped kinetic profile. These features suggest that the mechanism of transformation could be identical to that proposed in the previous section for mannitol and sorbitol but with a much faster metastability breaking (stage II), which nearly suppressed the incubation time. This behavior is expected to occur when the recrystallization of transient amorphous fractions is much less sensitive to the structural state of the surroundings crystallites so that it occurs preferentially towards the final form, α.

**Figure 4:**
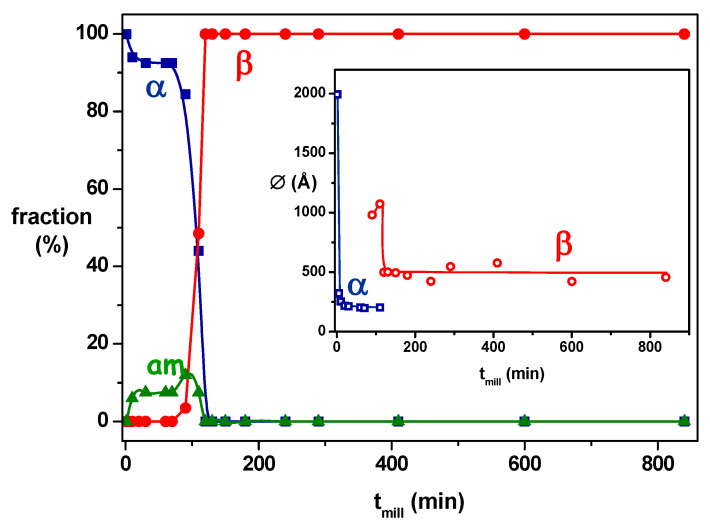
Kinetics of γ→α transformation of Bezafibrate under milling (black circles) and evolution of the size of crystallites of the γ (blue) and α (red) forms during milling, T_mill_, at the top. Evolution over time of the fraction of the different phases: α (blue circles), β (red circles), and amorphous (green triangles) of Bezafibrate and representation in insert of the size of crystallites α (blue squares) and β (red circles) at the bottom, according to [20]. The transformed fractions were derived from PXRD analysis. Reprinted with permission from [20]. Copyright 2022 American Chemical Society.

In the case of bezafibrate, the results of Dudognon et al. [20] highlighted that the milling of the commercial form α at 25 °C (15 °C below T_g_) led to the stable form β. The monitoring the transformation kinetics (Figure 4) reveals that it had the same appearance as that of monotropic systems (sorbitol [12] and mannitol [14]): a long induction time (several hours) followed by a rapid transformation stage (10 minutes). The polymorphic transformation is therefore likely to operate with the same mechanism.

**Figure 5:**
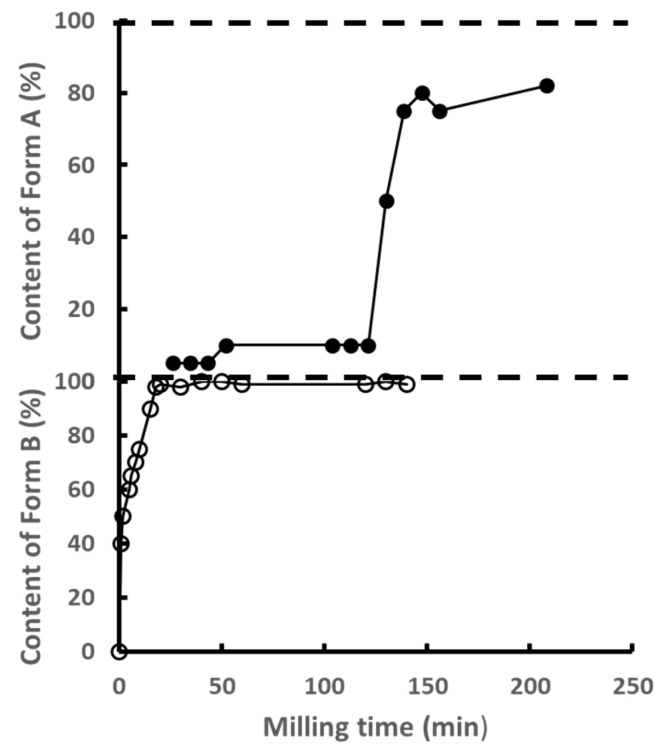
Kinetics of C→B (White circles) and B→A (Black circles) transformations of chloramphenicol palmitate under milling according to [70]. The transformed fractions were derived from PXRD analysis. Reproduced with permission from Chem. Pharm. Bull. Vol. 33 No.4. Pages 1660–1668 Copyright 1985 The Pharmaceutical Society of Japan.

Chloramphenicol palmitate [70] has more complex behavior upon milling, involving two successive polymorphic transformations, as shown in Figure 5. Interestingly, Kaneniwa et al. showed that the first transformation (C→B) involves the two metastable forms C and B, which are enentiotropically related. The corresponding kinetics resembles that of glycine as it has the shape of an exponential relaxation and do not show any sign of incubation. The second transformation (B→A) involves the metastable forms B previously produced by the milling and the stable form A, which are monotropically related. This second transformation starts nearly two hours after the completion of the first one and is completed in about 30 min. As a result, the transformation kinetics has a sigmoidal shape with a long incubation time. Moreover, during this incubation, a week stationary fraction of form A (~4%) could be detected revealing a mechanism of creation/annihilation of crystallites of form A, similar to that observed during the stage I of the transformation of mannitol (Figure 1). Chloramphenicol palmitate is thus particularly interesting and rich as it shows the two kinds of transformation kinetics (without and with incubation) between polymorphic forms, which are, respectively, enentiotropically and monotropically related.

Regarding enantiotropic materials, polymorphic transformations under milling can be observed from the metastable form to the stable one for Bezafibrate [20], Sulfamerazine [25], chloramphenicol palmitate [70], phenylbutazone [57], and caffeine [76,78]. The kinetic has a sigmoidal shape for Bezafibrate and chloramphenicol palmitate transformation B→A. On the other hand, there is only one enantiotropic material, glycine, that presents a phase transformation from the stable form to the metastable form. Given that for this material, the kinetics depends on the milling intensity, and that there is no proof of an intermediate amorphous form during the transition from the γ form to the α form, it was interesting to complete the work carried out on this compound. That is why we performed new experiments on glycine which are presented in the part II of this work.

Moreover, it would be necessary to study in more detail the kinetics of polymorphic transformation of other enantiotropic compounds to observe the existence or not of the induction time systematically observed in the case of monotropic transformations.

### 3.3. Detection of a Transient Amorphous Fraction During Polymorphic Transformations

A fundamental question remains whether the polymorphic transformations induced by milling occur through a direct solid–solid transformation or involve, on the contrary, a transient intermediate amorphization. Direct transformations are conceivable in the case of enantiotropic polymorphs, as transitions of the same nature are predicted by phase diagrams upon heating. Conversely, in the case of monotropic forms, such direct transformation appears less plausible, given that the corresponding transition does not exist in the phase diagram. From a practical standpoint, the formation of a transient amorphous phase is likely to be extremely short-lived, rendering its detection intrinsically challenging. This transient nature is generally attributed to the rapid recrystallization kinetics of the amorphous phase, resulting in a very short lifetime for each amorphous fraction generated by the mechanical impacts. Consequently, the instantaneous quantity of amorphous material present during milling is expected to be very weak and often below the detection limit of conventional X-ray diffraction techniques. However, for some compounds reported in Table 1, it was clearly demonstrated (directly or indirectly) that a partial amorphization of the initial crystalline form accompanied the polymorphic transformation towards the final form upon milling. This was particularly the case for sorbitol [12,13], Bezafibrate [20], Sulfamerazine [25], Sulfathiazole [39], ranitidine hydrochloride [45], rivastigmine hydrogen tartrate [46], indomethacin [57,58,59,60], and cimetidine [74].

**Figure 6:**
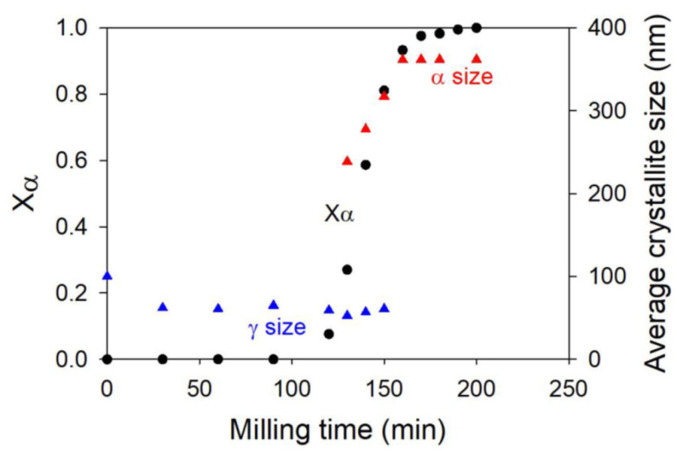
Experimental kinetics of γ→α transformation of sorbitol according to [12]. The left axis represents the fraction of α form. The right axis represents the average size of γ-form crystallites (in blue) and α-form crystallites (in red) according to [12]. The transformed fractions were derived from PXRD analysis. Reproduced from [12], with permission from [Elsevier], 2020.

For instance, a transient amorphization upon milling could be detected during the polymorphic transformation of some materials. It is the case, for instance, of sorbitol, where an indirect signature of a transient amorphization was revealed by the time evolution of the crystallite sizes of the initial and final forms. As shown in Figure 6, the average size of γ crystallites rapidly decreased during the first few minutes of milling to reach a stationary value around 50 A. On the other hand, the size of α crystallites rapidly increased after the induction time to finally reach 350 A, which was seven times larger than the stationary size of γ crystallites. Such a larger size of crystallites of the final form required a redistribution of matter, which was attributed to a transient amorphization of crystallites after impacts. The same behavior was observed in mannitol [14].

**Figure 7:**
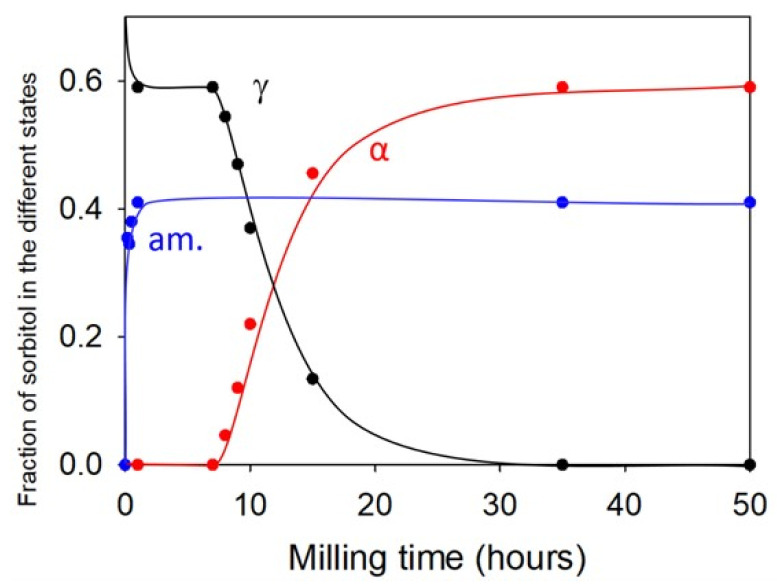
Evolution of the structural composition of sorbitol between the α (red), γ (black), and amorphous (blue) forms during milling according to [13]. The solid lines are guides for the eye. The transformed fractions were derived from PXRD analysis. Reproduced from [13], with permission from [Elsevier], 2022.

Dupont et al. [13] also gave a more direct evidence of this transient amorphization during the polymorphic transformation upon milling through co-milling experiments involving amorphous hydrochlorothiazide (HCT) and crystalline sorbitol in the form γ. In that case, the co-milling was found to produce a stationary heterogeneous material made of an amorphous molecular alloy hydrochlorothiazide/sorbitol, coexisting with crystalline sorbitol in the form α. The kinetic evolution of the different structural components during the milling followed by powder X-ray diffraction is reported in Figure 7. The most striking feature of this kinetics is that the polymorphic transformation γ→α of sorbitol only started after the complete formation of the amorphous molecular alloy component. This feature thus clearly indicates that the first effect of milling on sorbitol is an amorphization. This amorphization was here detectable because sorbitol combined with HCT to form a molecular dispersion with a high glass transition temperature. However, this T_g_ progressively dropped as the plasticization by sorbitol increased so that the stabilization effect stopped when the T_g_ of the molecular dispersion approached T_mill_. At this point, the newly amorphized fraction of sorbitol recrystallized towards the form α, leading to an apparent polymorphic transformation γ→α of the remaining γ form of sorbitol. These results thus definitely prove that in the case of sorbitol, the polymorphic transformation induced by milling involves a transient amorphization of the material. It must be noted that the glass transition temperatures of sorbitol and mannitol (T_g_ = −3 °C et T_g_ = 13 °C, respectively, see Table 1) are located well below T_mill_. As a result, the molecular mobility in the amorphous fraction is very high, and their recrystallization is very rapid, making its detection very difficult. That is why this amorphous fraction can only be detected indirectly.

Another interesting example is phenylbutazone [57], which is in the same situation as sorbitol (T_g_ = 4 °C). The authors did not directly observe the amorphous form of the compound during milling at 4° and 35 °C. However, the presence of a transient amorphous is the simplest way to describe the observations made on this material, of which the glass transition temperature is 4 °C. Let us recall that the authors made the following observations: During milling at 4 °C, the α, β, and δ forms were transformed into the ε form. During milling at 35 °C, the α and β forms were converted into the δ form, while the δ form remained in this form. This is consistent with the fact that upon impact, the material was amorphized, regardless of the initial form. The glass transition temperature of the material was low enough to directly obtain a recrystallization of this amorphous form. At 4 °C, the amorphous recrystallized in the ε form because this is the stable form at this temperature, as mentioned in reference [57]. At 35 °C, it recrystallized in the δ form for the same reason: δ form is the stable form at this temperature [57].

In certain cases, the transient formation of a small amorphous fraction could be observed directly during milling-induced polymorphic transformations. As illustrated in Figure 4 for Bezafibrate [20], the structural evolution of the compounds during the milling of the γ form demonstrated this phenomenon. A significant amorphous fraction (~10%) emerged rapidly within the first 100 min of milling, preceding the appearance of the β form. This amorphous content then decreased quickly, indicating recrystallization into the β form. This behavior revealed a competition between an amorphization of the material and a recrystallization which occurred towards the initial form α during the first hundred minutes of milling then towards the form β thereafter. It is thus perfectly in line with the four-stage transformation model proposed for mannitol in Section 3.1. Similar results have been obtained on Fananserin [16], ranitidine hydrochloride [45], and rivastigmine hydrogen tartrate [46]. For example, in the works of Amaro et al. [46], the observation of the diffraction patterns indicated that during the first hour of milling, the metastable Form II did not transform but became partially amorphous. The pattern recorded after two hours of milling indicated the appearance of Form I. After three hours, Form II was completely transformed into Form I, and no more “halo” characteristic of the amorphous form of the material was observed.

It should be noted that the glass transition temperatures of the five previous materials were, respectively, 19, from 13 to 30, 38, and 40 °C (see Table 1). These temperatures were close to the room temperature where the milling was performed so that the molecular mobility in the amorphous fraction was quite weak. In these conditions, the recrystallization was quite slow, which facilitated the detection of the amorphous fraction. Moreover, for long milling, the mechanical chocs of the balls progressively brought the temperature of the milling jar above T_g_, so that the mobility in the amorphous fraction became sufficient to observe its complete recrystallization towards the final form.

**Figure 8:**
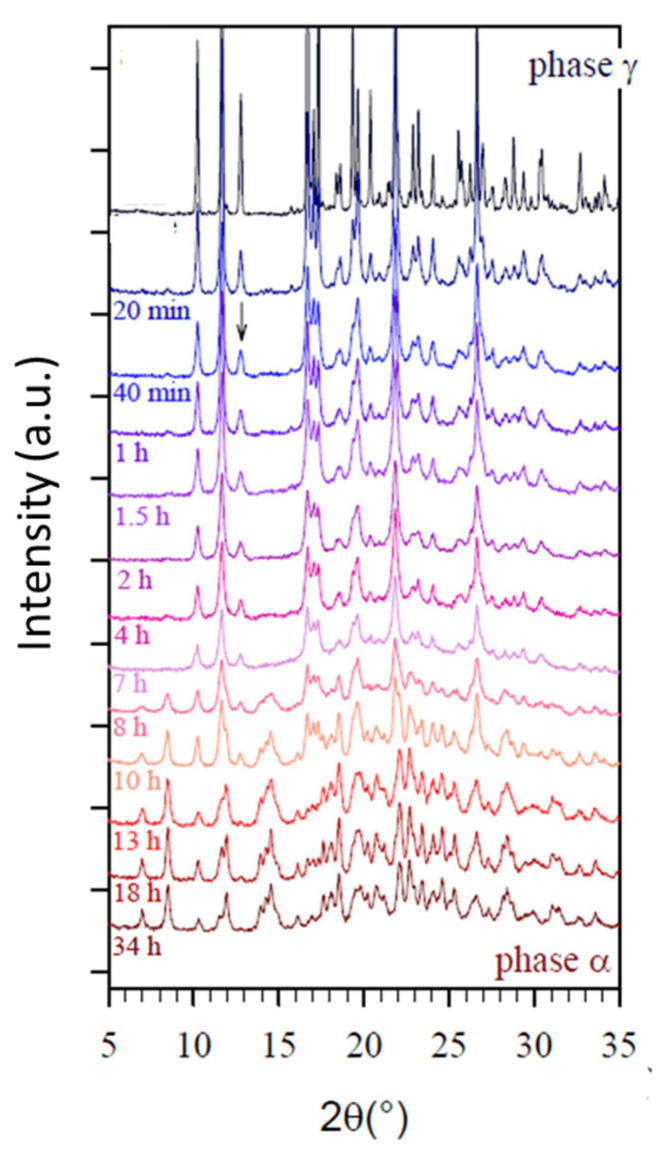
Evolution of diffractograms after milling of the γ form of indomethacin at room temperature according to [60]. Reproduced with the permission of Dr. Sylvain Desprez.

In the case of Sulfamerazine (form I) [25], Sulfathiazole (form II–V) [40], indomethacin (form γ) [60], and cimetidine (form B and C) [74], it was shown that milling leads to a stationary state where a new mixed polymorphic form and a noticeable amorphous fraction coexist. For instance, the milling of the metastable Form I of Sulfamerazine [25] produces a mixture of amorphous form and stable Form II. For Sulfathiazole [40], it was noted that a significant amount of amorphous form was generated during the milling-induced transformation. For indomethacin, as can be seen for example in Figure 8 from reference [60], and as was noted by the author of the work, the milling of the γ phase led to a decrease in the intensity of the Bragg peak and to the appearance an underlying broad diffusion halo reflecting the formation of an amorphous fraction in the samples. The author also notes that after 7 h of milling, the appearance of the Bragg peak of the α phase was observed. After 10 h of milling, the lines of the γ phase had completely disappeared, indicating that indomethacin was in the α form. In parallel with the disappearance of the γ phase, a decrease in the amorphous phase was observed which was, however, not complete. For cimetidine [74], a transition from metastable forms B and C to a mixture of Form I and amorphous form was observed. Interestingly, the glass transition temperatures of these four compounds are, respectively, 62, 67, 47 °C, and 43 °C, i.e., well above room temperature, so that the transient amorphous fraction generated during the milling has a low molecular mobility and is quite reluctant to crystallization. This explains why a high fraction of amorphous material can be easily detected during the milling and can even persist after the end of the milling.

To summarize, the work carried out in the literature on a certain number of compounds makes it possible to relate the T_g_ of the material, and the milling temperature, to the detection of transient amorphous and to the mechanism described in part III.1. Initially, the impacts of balls on the material reduce the crystallite sizes and cause its amorphization. This first point is well established in the case of amorphizations under milling [15,28,86]. The analysis carried out here of the different compounds in Table 1 shows that the speed of the amorphization–recrystallization mechanism then depends on the relative positions of T_g_ and T_mill_. The most extreme case, where T_g_ is much higher than T_mill_, is well known; it is the one where the material is totally amorphized without ever recrystallizing [15,28,86].

The different compounds listed in Table 1 show that when T_g_ approaches T_mill_, then the molecular mobility of the amorphous material allows its partial recrystallization. This is what has been observed in the case of Sulfamerazine (T_g_ = 62 °C) [25], Sulfathiazole (T_g_ = 67 °C) [40], indomethacin (T_g_ = 47 °C) [57,58,59,60], and cimetidine (T_g_ = 43 °C) [74]. When T_g_ becomes even closer to room temperature, the amorphous material recrystallizes completely to a crystalline form, but slowly enough for it to be detectable during milling. This is what has been observed for Bezafibrate (T_g_ = 40 °C) [20], ranitidine hydrochloride (13 < T_g_ < 30 °C) [45], rivastigmine hydrogen tartrate (T_g_ = 38.2 °C) [46], and Fananserine (T_g_ = 19 °C) [16].

Finally, when T_g_ is well below room temperature, the molecular mobility is such that the amorphization/recrystallization process is very rapid and leads to the observation of an A→B transformation, apparently direct, during milling. This is what has been proven on sorbitol (T_g_ = −3 °C) [12,13] and which explains the observations made on phenylbutazone (T_g_ = 4 °C) [57]. Further analyses, similar to those carried out for sorbitol, were necessary to extend this behavior to the mannitol [14].

## 4. Conclusions

The effect of milling on a material can thus be summarized as follows:

- Total and long-lasting amorphization occurs when the milling is permed below the T_g_ of the material gives rise to a crystal to glass transformation.
- Transient amorphization is followed by a more or less rapid recrystallization when the milling is performed above T_g_. If this recrystallization occurs towards the starting polymorphic form, no apparent structural change is observed. On the other hand, if the recrystallization occurs towards another polymorphic form, an apparent polymorphic transformation is observed. The nature of the form which recrystallizes can hardly be anticipated as one or several metastable phases can develop as predicted by the Ostwald’s rule of stages [87]. However, in practice, the recrystallized form is often identical to that obtained for the recrystallization of the corresponding quenched liquid upon heating.

In this review, 18 pharmaceutical compounds exhibiting a polymorphic trans-formation under milling were listed, and the essential characteristics of the transformations were analyzed to figure out the mechanism of these transformations. In particular, the following parameters were studied: the T_g_ of the material and its position relative to the milling temperature, the kinetics of the polymorphic transformation, the detection or not of an intermediate or coexisting amorphous form, the stable or metastable character of the final and starting forms, and finally, the relationships between these forms (monotropic or enantiotropic). The elements studied and the experiments complementing this work, published in part II, allow the generalization, to a certain extent, of the polymorphic transformation mechanism described in the context of sorbitol and mannitol. This mechanism is based on a rapid amorphization of the starting compound and a more or less rapid recrystallization towards the final form. Recrystallization towards the final form depends on the accidental formation of a cluster of this form during milling, which explains the induction time observed almost systematically in the kinetics of milling induced transformation. Recrystallization velocity also depends on the position of T_g_ relative to the T_mill_, which explains why the amorphous form of the compound may not be detected (T_mill_ higher or much higher than T_g_) or, on the contrary, coexist with the final form (T_g_ higher or around T_mill_).

To generalize this model and to confirm the existence of the different steps described here in the process of polymorphic transformations under milling, it would be necessary to complete the work described here. Therefore, in part II of this publication, additional work was carried out on mannitol, famotidine, Sulfamerazine, and glycine, which all exhibit polymorphic transformations under milling.

## Figures and Tables

**Table 1:** List of pharmaceutical materials exhibiting polymorphic transformation and information relating to these transformations.

Pharmaceutical Materials and Molecular Weight	Stating Form and Lattice Parameters (Å)	Final Form and Lattice Parameters (Å)	Relationship	Kinetic Transformation Curve Shape	Information on the Polymorphic Transformation	T_g_ (°C)	Melting Temperature (°C)	Ref
Sorbitol (C_6_H_14_O_6_) 182.17 g/mol	γ a = 24.301 b = 20.572 c = 4.867 α = β = γ = 90°	α a = 9.048 b = 4.870 c = 18.262 α = β = γ = 90°	Monotropic	Sigmoidal	Transformation from the stable γ form to the metastable α form through a transient amorphous phase	−3	95	[12,13]
Bezafibrate (C_19_H_20_ClNO_4)_ 361.82 g/mol	α a = 10.3118 b = 17.6601 c = 19.7133 α = β = γ = 90°	β a = 10.7849 b = 15.7886 c = 11.4932 α = γ = 90° β = 115.875°	Enantiotropic	Sigmoidal	Transformation from the α form (stable at high temperature) to the β form (stable at room temperature) through a transient amorphous phase	40	175	[20]
Sulfamerazine (C_11_H_12_N_4_O_2_S) 264.30 g/mol	I a = 14.474 b = 21.953 c = 8.203 α = β = γ = 90°	II a = 9.145 b = 11.704 c = 22.884 α = β = γ = 90°	Enantiotropic	Unknown	Transformation from form I (stable at high temperature) to a mixture of amorphous phase and form II (stable at room temperature)	62	237	[25]
Mannitol (C_6_H_14_O_6)_ 182.17 g/mol	β a = 5.5381 b = 8.580 c = 16.795 α = β = γ = 90°	α a = 4.8653 b = 8.873 c = 18.739 α = β = γ = 90°	Monotropic	Sigmoidal	Transformation from the stable β form to the metastable α form	13	166	[14]
Glycine (C_2_H_5_NO_2_) 75 g/mol	γ a = b = 7.035 c = 5.481 α = β = 90° γ = 120°	α a = 5.107 b = 12.040 c = 5.460 α = γ = 90° β = 111.82°	Enantiotropic	Depends on the milling intensity	Transformation from the γ form (stable at room temperature) to the α form	Unknown	Unknown	[11,82]
Sulfathiazole (C_9_H_9_N_3_O_2_S_2_) 255.31 g/mol	V a = 10.399 b = 15.132 c = 14.280 α = γ = 90° β = 1.21° IV a = 10.867 b = 11.456 c = 8.543 α = β = 90° γ = 91.87° III a = 17.570 b = 8.574 c = 15.583 α = γ = 90° β = 112.93° II a = 8.235 b = 8.550 c = 15.558 α = γ = 90° β = 93.67°	I a = 10.554 b = 13.220 c = 17.050 α = γ = 90° β = 108.06° I I I	Unknown	Unknown	Transformation from form II, III, IV, and V to a mixture of amorphous phase and form I	67	200	[40,41]
Ranitidine Hydrochloride (C_13_H_22_N_4_O_3_S·HCl) 350.86 g/mol	I a = 12.1918 b = 6.5318 c = 22.0382 α = γ = 90° β = 93.985°	II a = 18.798 b = 12.980 c = 7.204 α = γ = 90° β = 95.09°	Monotropic	Sigmoidal	Transformation from form I (metastable) to form II (stable) through a transient amorphous phase	13–30	I→134–140 II→140–144	[44,45]
Rivastigmine hydrogen tartrate (C_14_H_22_N_2_O_2_·C_4_H_4_O_6)_ 400.42 g/mol	II a = 17.538 b = 8.326 c = 7.261 α = γ = 90° β = 98.799°	I Unknown	Monotropic	Induction time of 1 h	Transformation from form II (metastable) to form I (stable) through a transient amorphous phase	38.2	II→97.4 I→124.5	[46]
Famotidine (C_8_H_15_N_7_O_2_S_3_) 337.44 g/mol	B a = 17.057 b = 5.335 c = 17.776 α = γ = 90° β = 116.6°	A a = 11.986 b = 7.200 c = 16.818 α = γ = 90° β = 99.82°	Monotropic	Unknown	Transformation from form B (metastable) to form A (stable)	50	165	[50,51]
Gabapentin (C_9_H_17_NO_2_) 171.24 g/mol	I a = 14.567 b = 9.2153 c = 7.6503 α = γ = 90° β = 93.375° II III	II a = 5.8759 b = 6.9198 c = 22.262 α = γ = 90° β = 90.080° III a = 30.5452 b = 5.9268 c = 10.8841 α = γ = 90° β = 108.316° IV a = 14.537 b = 6.633 c = 9.834 α = γ = 90° β = 105.92° IV	Unknown	Unknown	Transformation from form I to form II Transformation from form II to a mixture of form III and IV Transformation from form III to form IV	Unknown	166	[52]
Indomethacin (C_19_H_16_ClNO_4_) 357.79 g/mol	γ a = 9.2173 b = 9.6060 c = 10.8436 α = 69.959 β = 87.1970 γ = 69.501	α a = 5.4616 b = 25.310 c = 18.152 α = γ = 90° β = 94.38°	Monotropic	Sigmoidal	Transformation from the γ form (stable) to the α form (metastable) through a transient amorphous phase	47 °C	163	[59,60,62]
Modafinil (C_15_H_15_NO_2_S) 273.35 g/mol	I a = 14.5022 b = 9.6875 c = 20.8445 α = γ = 90° β = 110.17° IV a = 18.172 b = 52.375 c = 5.698 V Unknown VI Unknown	III a = 14.510 b = 9.710 c = 19.569 α = β = γ = 90° III III III	Monotropic	Unknown	Transformation from form I, IV, V, and VI (metastable) to the III (stable)	43 °C	165	[9]
Fananserin (C_23_H_24_FN_3_O_2_S) 425.52 g/mol	III a = 14.625 b = 14.370 c = 720.356 α = γ = 90° β = 92.84° IV a = 8.633 b = 9.714 c = 12.270 α = γ = 90° β = 96.70°	I a = 8.359 b = 17.228 c = 8.089 α = 101.32 β = 110.85 γ = 86.85 I	Monotropic	Induction time of more than 1 h for the IV→I transformation	Transformation from form III (metastable) and IV (stable) to form I (metastable) through a transient amorphous phase	19	III→101 IV→99	[16]
Chloramphenicol Palmitate (C_27_H_42_Cl_2_N_2_O_6_) 561.54 g/mol	C Unknown B Unknown	B Unknown A a = 7.805 b = 52.503 c = 7.414 α = β = γ = 90°	Enantiotropic Monotropic	Exponential Relaxation Sigmoidal	Transformation from form C (metastable) to form B (metastable) then to form A (stable)	Unknown	C→B à 64.5 A→90.3 B→86.7	[69]
Cimetidine (C_10_H_16_N_6_S) 252.34 g/mol	B a = 55.45 b = 5 c = 18.72 α = γ = 90° β = 100.4° C a = 82.904 b = 4.85 c = 18.760 α = γ = 90° β = 74.34°	A a = 10.7029 b = 18.8262 c = 6.8266 α = γ = 90° β = 111.306° A	Monotropic	Unknown	Transformation from form B and C (both metastable) to form A (stable) through a transient amorphous phase	43	A→140–152 B→142–145 C→145–154	[74,75]
Phenylbutazone (C_19_H_20_N_2_O_2_) 308.37 g/mol	4 °C Milling α a = 21.415 b = 5.7295 c = 27.782 α = γ = 90° β = 108.4° ß Unknown δ Unknown 35 °C Miling α ß δ	4 °C Milling ɛ Unknown ɛ ɛ 35 °C Milling δ δ δ	ß/δ Monotropic α/ß Enantiotropic α/δ Enantiotropic	Unknown	At 4 °C: transformation from the α, β, and δ form to the ε form after several hours of milling At 35 °C: transformation from the α and β form to the δ form after several hours of milling	4	α→91.2 ß→93.3 δ→101.4	[76,77,83]
Nolomirole Hydrochlorride (C_19_H_28_ClNO_4_) 369.88 g/mol	α Unknown	β Unknown	Unknown	Unknown	Transformation from the α form to the β form	Unknown	Unknown	[78]
Caffeine (C_8_H_10_N_4_O_2)_ 194.19 g/mol	I a = 14.9372 b = 14.9372 c = 6.8980 α = β = 90 γ = 120 II	II a = 43.0390 b = 15.06758 c = 6.95314 α = γ = 90° β = 99.0274° I	Enantiotropic	Transformation too fast to observe kinetics	Transformation from form I (metastable) to form II (stable) Transformation from form II (stable) to form I (metastable)	−17	227	[79,81,84]
